# Strawberry Leaf Extract Treatment Alleviates Cognitive Impairment by Activating Nrf2/HO-1 Signaling in Rats With Streptozotocin-Induced Diabetes

**DOI:** 10.3389/fnagi.2020.00201

**Published:** 2020-07-23

**Authors:** Li Zhang, Qinghua Ma, Yanling Zhou

**Affiliations:** ^1^Department of Nursing, The People’s Hospital of Suzhou New District, Suzhou, China; ^2^Department of Prevention and Health Protection, The 3rd People’s Hospital of Xiangcheng District, Suzhou, China; ^3^Department of Operation, The Second Affiliated Hospital of Soochow University, Suzhou, China

**Keywords:** cognitive decline, strawberry leaf extract, apoptosis, inflammatory cytokine, oxidative stress, Nrf2-HO-1 signaling

## Abstract

Diabetes-associated cognitive impairment (DACI) is a common complication of diabetes mellitus (DM) that affects the central nervous system. Cognitive impairment, such as learning and memory impairment, and even dementia, is the main clinical manifestation of DACI. Unfortunately, there is no effective means by which to delay the cognitive symptoms of DM. Evidence has shown that strawberry leaf extract could alleviate cognitive decline, suppress oxidative stress, and reduce inflammatory responses in rats. In the present study, we evaluated the effect of strawberry leaf extract on cognitive function in a rat model of streptozotocin (STZ)-induced diabetes. After the continuous administration of strawberry leaf extract for 4 weeks, the Morris Water Maze (MWM) test results showed that the cognitive impairment of the rats was alleviated. Moreover, strawberry leaf extract significantly reduced the level of reactive oxygen species (ROS), decreased the level of malondialdehyde (MDA), improved the activity of superoxide dismutase (SOD) and catalase (CAT), decreased the mRNA expression of interleukin-6 (IL-6) and tumor necrosis factor-α (TNF-α) and decreased the protein expression of caspase-3 and caspase-9 in the hippocampus of DM rats. Also, transcription factor nuclear factor erythroid 2 (NF-E2)-related factor 2 (Nrf2)/hemeoxygenase-1(HO-1) signaling was activated by the administration of strawberry leaf extract. Our findings indicate that strawberry leaf extract may be a potential drug candidate for the treatment of DACI and may be used as a basis for further research on the development of drugs for cognitive impairment in diabetes.

## Introduction

Diabetes mellitus (DM) is a metabolic disease commonly observed in the clinic and is characterized by hyperglycemia due to impaired insulin secretion, insulin resistance, or both, resulting in impaired metabolism of sugars, lipids, and proteins. DM is associated with the peripheral nervous system and central nervous system complications.

Diabetic cognitive dysfunction is considered to be an important complication of diabetes that affects the central nervous system (Devaskar et al., 1994; Kuwabara et al., 2011). DM is an independent risk factor for cognitive dysfunction, and the degree of cognitive decline is related to the course of DM (Ebady et al., 2008). A previous study found that the risk of cognitive decline in middle-aged diabetic patients is 2–3 times higher than that in nondiabetic patients of the same age (Luchsinger et al., 2007; Nooyens et al., 2010). Cognitive deficit is a common but underestimated complication of DM that can cause learning and memory dysfunction (Koekkoek et al., 2015). The impairment of cognitive function is characterized by a decline in memory, language, and the ability to process complex information, while the decline in the basic attention process, motor reaction time, and transient memory may be relatively small (Venkat et al., 2019). The mechanism of diabetes-associated cognitive impairment (DACI) is not entirely clear. There is a widespread view that hyperglycemia and insulin resistance are closely related to DACI. Additionally, it has been consistently suggested that oxidative stress and inflammatory cytokines seem to be strongly associated with DACI (Emerit et al., 2004; Caspersen et al., 2005; Marioni et al., 2010; Hwang et al., 2014). Anti-inflammatory processes and pro-inflammatory processes antagonize each other, increasing oxygen consumption, and producing a large amount of oxygen free radicals. hyperglycemia may lead to excessive oxidative stress production and mitochondrial impairment, which promotes the progression of DACI (Kumar et al., 2009). Antioxidant drugs may slow the progression of DACI.

Leaves and fruits, which are rich in antioxidants and bioactive ingredients, can help protect humans from many chronic diseases (Upadhyay and Mohan Rao, 2013). The strawberry (Fragaria × ananassa Duch) is native to South America, and it is widely cultivated in China and Europe. The leaves of strawberries are rich in tannic acid, ascorbic acid, and other bioactive compounds (Upadhyay and Mohan Rao, 2013). It has been reported that ascorbic acid is considered to be an antioxidant that has been proven to counteract the harmful effects that are related to reactive oxygen species (ROS)-induced injury (Muripiti et al., 2019). Strawberry leaves contain carotene and vitamin A, which can alleviate night blindness, maintain healthy epithelial tissues, improve eyesight, and promote growth and development. Strawberry leaves are also used to treat a series of other conditions such as anemia, nervous problems, and arthritis. Studies have shown that Fragaria nilgerrensis Schlecht extract can regulate blood sugar and improve the memory dysfunction induced by STZ (Gao et al., 2018). Usually, it is believed that the Nrf2/HO-1 signaling pathway plays an important role in oxidative stress (Espada et al., 2010). Also, Fan et al. (2012) have found that high expression of HO-1 can resist apoptosis, inflammatory and oxidative stress in the cerebral cortex and retina of type 1 diabetic rats induced by STZ, and the expression of HO-1 is closely related to Nrf2. In this study, we evaluated the effects of strawberry leaf extract (SL) on learning and memory function, oxidative stress production inflammatory cytokines, and apoptosis-related protein expression in rats with STZ-induced diabetes. To further explore the underlying mechanism by which strawberry leaf extract controls DACI, we also assessed Nrf2/HO-1 signaling.

## Materials and Methods

### Preparation of Strawberry Leaf Extract

Strawberry leaves were obtained in March 2018 from Suzhou, China. Ripe and healthy strawberry leaves were collected in the morning and sent to the laboratory. The strawberry leaves were naturally dried at a constant temperature of 20°C in the dark and then ground into powder. One-hundred grams of the powder was dissolved in 1,000 ml distilled water for 1 h, air-dried (20°C in the dark), filtered, and vaporized in a rotary evaporator to obtain a residue. The yield of crude residue was 55%.

### Experimental Animals

Forty male 10-week-old Sprague–Dawley (SD) rats were purchased from the Laboratory Animal Center of Soochow University (Suzhou, China), housed at a temperature (24 ± 2°C) with a 12 h light/dark cycle, and given sufficient food and water. All of these procedures were conducted to ease the suffering of the animals and reduce the number of experimental animals. The experiment was conducted following the ethics committee of Soochow University. A diabetic rat model was induced by a single intraperitoneal injection of STZ (50 mg/kg, Abcam, ab142155, UK; Medras et al., 2017). After 72 h, blood was collected from the tail vein to test the blood glucose of all the animals. Blood glucose >200 mg/dl indicated the successful establishment of the model in the rats. The animals were randomly assigned to four groups as follows: control group (CON group), CON + Strawberry Leaf group (CON + SL group) DM group, and DM model + Strawberry Leaf group (DM + SL group), with 10 rats in each group. The rats in the Con and DM groups were given equal volumes of distilled water. The other diabetic rats received strawberry leaf extract (SL) treatments at a dose of 200 mg/kg (Erfani Majd et al., 2019) once a day for 4 weeks.

### FPG Measurements

Blood was collected from the tail vein after 4 weeks of strawberry leaf administration and assessed using Ibrahim and El-Maksoud’s (2015) method.

### New Object Recognition (NOR) Test

The experimental device was a gray square with a bottom surface of 40 cm × 40 cm, which was surrounded by walls, with a height of 45 cm. Twenty-four hours before the test or training, the animal was placed in the test room and adapted to the test environment. First, two objects (A and B) were placed in the experimental device. The objects A and B were identical. The two objects A and B were placed on the left and right ends of a sidewall, and the rat was placed with its back facing the two objects in the field; the distance of the nose of the rat from the two objects was the same. Immediately after placement, the recording equipment was turned on, and the contact between the rat and the objects, including the number of times the rat’s nose or mouth touched the objects and the time of exploration within 2–3 cm of the object, was recorded. After 10 min, the rat was immediately returned to its box in the test room. After the rat had rested for 1 h, the official test began. At this time, object B was replaced with a new object C, and the investigation of object C by the rat was observed for 2–5 min. The discrimination index was calculated based on the observation index: the discrimination index = (N − F)/(N + F), where “N” means the animal’s exploration time (s) of the novel object (C) during the test period, and “F” means the animal’s exploration time (s) of the familiar object (B) during the test period.

### Morris Water Maze Test

After 4 weeks of treatment, the learning and memory abilities of all the animals were assessed using the Water Morris Maze (WMW) test. The WMW equipment was a round container, 150 cm in diameter and 70 cm in height, filled with room temperature water. A platform with a diameter of 10 cm was placed under 2 cm of water. All the experimental animals were tested for 5 days. One to four days of the experiment, the rats were placed into water and given 60 s to find and climb onto the platform hidden below the water. The rats that failed to find the platform within 60 s were guided to it and allowed to stay on the platform for 10 s. On day 5 of the test, the platform was removed, and the rats were allowed to swim in the pool. The time spent in the target quadrant was recorded as a parameter of learning and memory.

### Hematoxylin-Eosin (HE) Staining

To assess the morphological changes of the hippocampal neurons in the rats, brain tissue was harvested after the animals were sacrificed. The tissue was fixed in 4%paraformaldehyde for 48 h and embedded in paraffin. The blocks of wax were sliced along the coronal plane and stained with hematoxylin-eosin to observe the neurons under an optical microscope.

### Analysis of Cytokines and Oxidative Stress Products in the Hippocampus

The hippocampal samples were washed with cold normal saline and then homogenized on ice. The prepared homogenate was centrifuged for 10 min at 3,000 r/min to obtain supernatants. Interleukin-6 (IL-6), tumor necrosis factor-α (TNF-α), interleukin-4 (IL-4), and interleukin-10 (IL-10) were detected by ELISA kits [Rat TNF alpha ELISA Kit (RAB0480) Roche, Switzerland; Rat IL-6 ELISA Kit (RAB0278) Roche, Switzerland; Rat IL-4 ELISA Kit (ab100771), Abcam; Rat IL-10 ELISA Kit (100764), Abcam]. The MDA and superoxide dismutase (SOD) activities were determined by colorimetric assay Kits. A catalase (CAT) assay kit and a ROS assay kit were used to detect the activity of CAT and the level of ROS. SOD, MDA, CAT, and ROS assay kits were purchased from Nanjing Jiancheng Bioengineering Institute. All the experimental processes were performed following the manufacturer’s instructions.

### Real-Time PCR Analysis

Total RNA was extracted from the hippocampus using TRIzol Reagent (Invitrogen, USA), and then quantitative real-time reverse-transcriptase PCR was performed using the Bio-Rad thermocycler and SYBR green kit (Invitrogen, USA). The relative mRNA levels were normalized to β-actin as an internal standard. The following sequence-specific primers were used: IL-6 5′-CACACGTCGGGAGAGG-AGAC-3′ and 5′-ACAGTGGATCATCGCTCTTC-3′, TNF-α 5′-GAGAGACCGGCTGCTGGAAC-3′ and 5′-TGGAGATTATGATGACCGTA-3′, IL-4 5′-CTCAGGACGGTAAGGTTCAATG-3′ and 5′-GTTTACCCGCCGATGTTGTCG-3′, IL-10 5′-CCCTTCCATACTCCACGTTGG-3′ and 5′-TAATAGCCCTGGCACCTCGGC-T-3′, and β-actin 5′-CGCATCACACCTTCTAC-3′ and 5′-CTGG-CTCATCTTGGCAC-3′.

### Western Blot Detection

The protein expression of Nrf2, HO-1, caspase-3, and caspase-9 was detected by Western blot. Five rats in each group were selected and sacrificed under deep anesthesia. The hippocampal tissues were rapidly isolated on ice and stored in a freezer at −80°C. The hippocampus was homogenized with assay lysis buffer. The proteins were then separated by electrophoresis on 10% SDS-PAGE gels and transferred onto PVDF membranes. The membranes were blocked with 5% skimmed milk for 2 h at room temperature and then incubated with primary antibodies overnight at 4°C. The antibodies were as follows: Nrf2 (1:1,000, Abcam, USA), HO-1 (1:1,000, Abcam, USA), caspase-3 (1:2,000, Abcam, USA), caspase-9 (1:2,000, Abcam, USA). After several washes, the membranes were incubated with horseradish peroxidase-conjugated secondary antibodies for 1 h at room temperature. ImageJ software was used to quantify the protein bands. All the optical densities were normalized to that of β-actin (Tian et al., 2017).

The protein of the five animals per group pulled and used in the electrophoresis, and then selected a representative one. The results were the mean of five tests.

### Statistical Analysis

SPSS 23.0 software was used to analyze the data. The data are expressed as the mean ± SD. *p* < 0.05 was considered to indicate a statistically significant difference. The data were analyzed by one-way ANOVA, followed by Bonferroni *post hoc* correction. Repeated-measures ANOVA was used to assess the escape latency in the MWM, and days were considered repetitive factors.

## Results

### Strawberry Leaf Extract Improved the Blood Glucose

**Figure 1:**
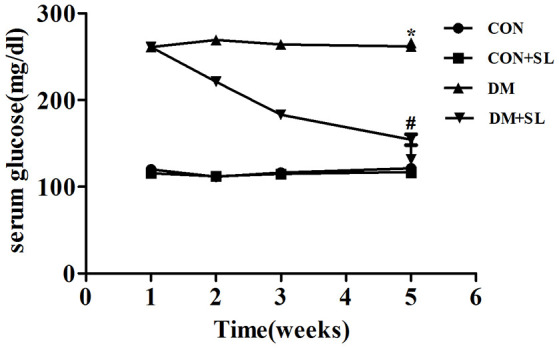
Effect of strawberry leaf extract on FPG. Data are shown as mean ± SD. *p < 0.05 vs. CON group; ^#^*p* < 0.01 vs. diabetes mellitus (DM) group, *n* = 10.

The blood glucose of diabetic rats was significantly higher than that of the normal control rats (*F* = 18.23, *p* < 0.05). After strawberry leaf extract administration, the blood glucose of the diabetic rats was significantly decreased (*F* = 13.27, *p* < 0.05; Figure 1). Our results showed that strawberry leaf extract could effectively control blood sugar.

### Strawberry Leaf Extract Suppressed the Learning and Memory Deficits Induced by Diabetes

**Figure 2:**
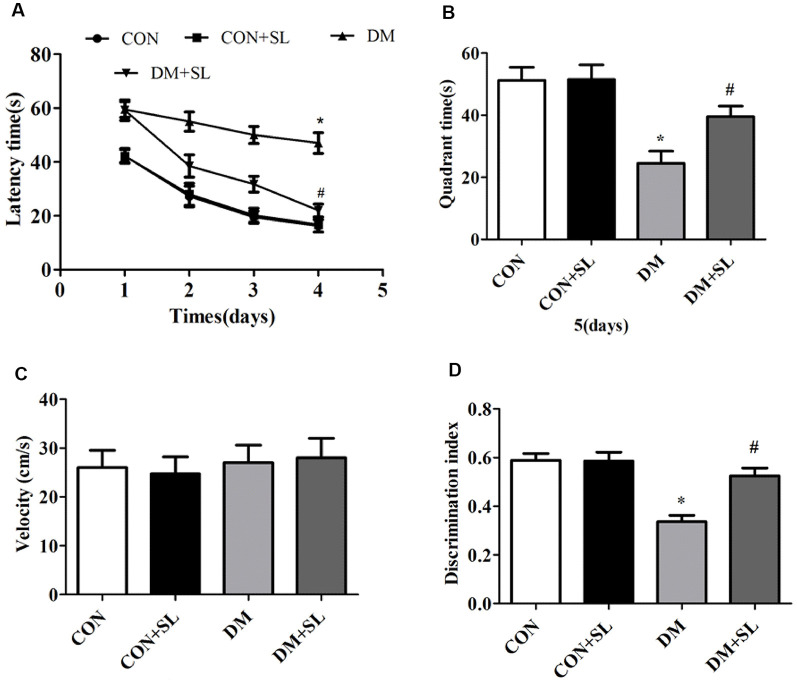
Effect of strawberry leaf extract on learning and memory of rats with streptozotocin (STZ) induced diabetes determined by the Morris Water Maze (MWM) and new object recognition (NOR) tests. **(A)** The performance of the rats in the orientation navigation tests was evaluated by the average escape latency of four trials on each of four consecutive days. **(B)** Time spent swimming in the target quadrant in the probe test. **(C)** Swimming speed of all the animals. **(D)** Rats performance in the new object experiments, using the discrimination index to evaluate the cognitive ability. The data in each group are shown as mean ± SD, using repeated-measures ANOVA with *post hoc* Bonferroni tests to determine the statistical significance. *p < 0.05 vs. CON group; ^#^*p* < 0.01 vs. DM group, *n* = 10.

Hippocampus-dependent cognitive ability was tested by the MWM hidden platform test. Figure 2A shows the effects of SL administration on learning and memory abilities as observed by latency trials. The rats in the DM group spent more time searching for the hidden platform than the rats in the CON group on day 2 (*F* = 19.75, *p* < 0.05), 3 (*F* = 18.45, *p* < 0.05) and 4 (*F* = 21.62, *p* < 0.05) of the orientation navigation tests. In the DM+SL group, SL administration significantly shortened the escape latency at day 2 (*F* = 42.55, *p* < 0.05), 3 (*F* = 34.23, *p* < 0.05) and 4 (*F* = 35.81, *p* < 0.05; Figure 2A). On day 5, in the probe test, the DM rats spent less time in the target quadrant than the CON rats (*F* = 33.38, *p* < 0.05). In the DM+SL group, after SL treatment, a marked improvement was observed (*F* = 27.26, *p* < 0.05; Figure 2B). Nevertheless, in the whole test, the swimming speed was not significantly different among all the rats (*F* = 2.43, *p* > 0.05; Figure 2C). Moreover, the difference in the discrimination index of the four groups was statistically significant (*F* = 18.13, *p* < 0.05). We found that the discriminatory index of the CON group and the SL group was higher than that of the DM group, and the difference was statistically significant (*F* = 21.05, *p* < 0.05); however the discriminatory index of the CON group was not significantly different from that of the CON+SL group (*F* = 1.63, *p* > 0.05; Figure 2D). Taken together, our findings indicated that diabetes could induce cognitive decline; however, SL could suppress the learning and memory deficits in the DM rats.

### Strawberry Leaf Extract Prevented Neuronal Cell Loss in the Hippocampal CA1 Region

**Figure 3:**
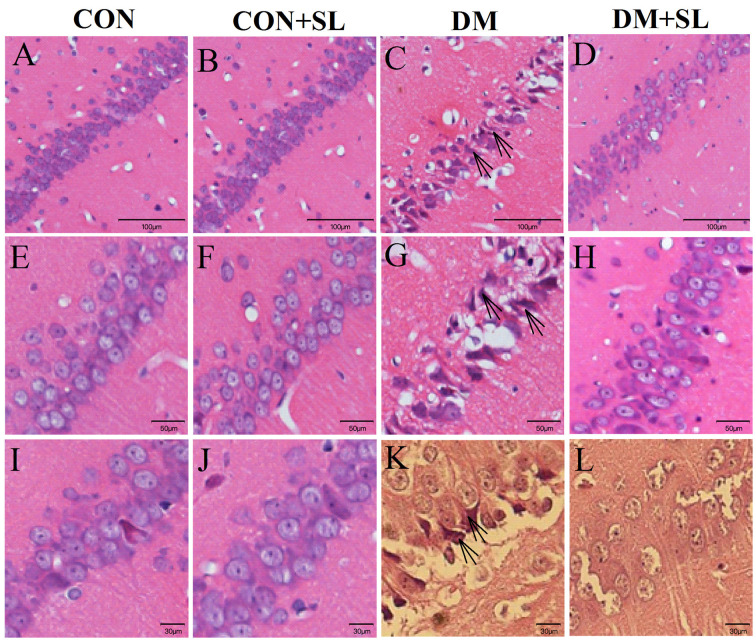
Morphological changes in the neurons in the CA1 hippocampal region of diabetic rats were observed under an optical microscope. Representative images of HE staining in brain slices of the hippocampus. **(A–D)** Magnification 4×, **(E–H)** Magnification 10×, **(I–L)** Magnification 40×. The arrow points to a positive sign (disorder arrangement, dilated intercellular spaces and karyopyknosis) *n* = 5.

Diabetes could reduce cognitive function in rats. Next, we observed the morphological changes of neurons in the hippocampal CA1 region in the rats using an optical microscope. The HE-stained hippocampal tissue sections were enlarged 4× (Figures 3A–D), 10× (Figures 3E–H), and 40× (Figures 3I–L). The normal hippocampal nuclei were large, round, and lightly stained (Figures 3A,B,E,F,I,J). In the DM group, many nuclei became shrunken and dark, which indicated that the cells were dying (Figures 3C,G,K). After SL treatment, the abovementioned cell death was significantly improved (Figures 3D,H,L). Based on the morphological observation of the rat hippocampus, the cognitive function of the diabetic rats may be related to the morphological changes of the hippocampal neurons, and SL could significantly alleviate hippocampal neuronal damage caused by diabetes.

### Strawberry Leaf Extract Changed MDA and ROS Levels and SOD and CAT Activities

**Figure 4:**
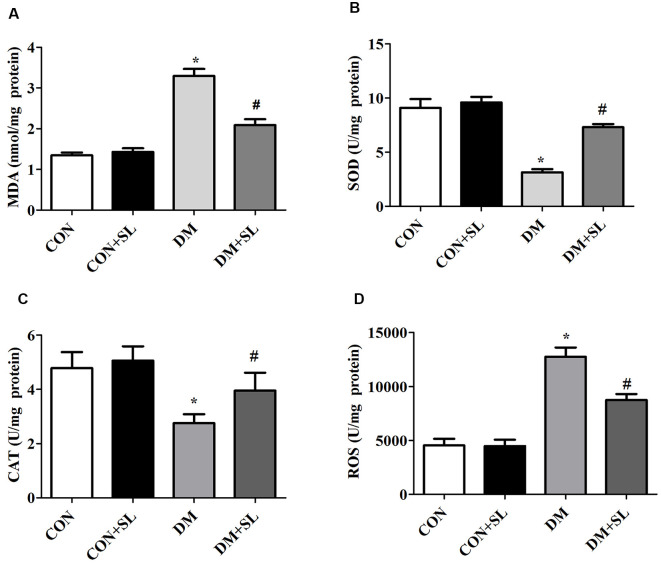
Effect of strawberry leaf extract on oxidative stress associated factors. **(A)** The level of MDA; **(B)** The activity of superoxide dismutase (SOD); **(C)** The activity of CAT. **(D)** The level of reactive oxygen species (ROS). The data in each group are shown as the mean ± SD. *p < 0.05 vs. CON group; ^#^*p* < 0.01 vs. DM group, *n* = 5.

Our results have shown that cognitive impairment in diabetic rats is closely associated with hippocampal neuromorphological changes. We evaluated the levels of oxidative stress in the rats in the four groups. The levels of ROS and MDA were determined by colorimetric assay kits. The data showed that diabetes caused a significant increase in the levels of MDA and ROS in rats (*F* = 14.47, *p* < 0.05, *F* = 16.81, *p* < 0.05). After 4 weeks of administration, the SL significantly reduced the levels of MDA and ROS in the rats of the DM group (*F* = 16.68, *p* < 0.05; *F* = 13.56, *p* < 0.05; Figures 4A,D). The antioxidant enzymes, SOD and CAT, were determined by colorimetric assay Kits. STZ-induced Diabetes significantly reduced the SOD and CAT activities in rats (*F* = 10.84, *p* < 0.05; *F* = 14.18, *p* < 0.05). After 4 weeks of administration, the strawberry leaf extracts significantly enhanced the activity of SOD and CAT in the rats of the DM group (*F* = 16.69, *p* < 0.05; *F* = 15.23, *p* < 0.05; Figures 4B,C). The experimental results have demonstrated that cognitive dysfunction in diabetic rats may be related to oxidative stress injury, and SL can improve diabetic cognitive dysfunction by inhibiting oxidative stress.

### Strawberry Leaf Extract Suppressed DM-Induced Neuroinflammation

**Figure 5:**
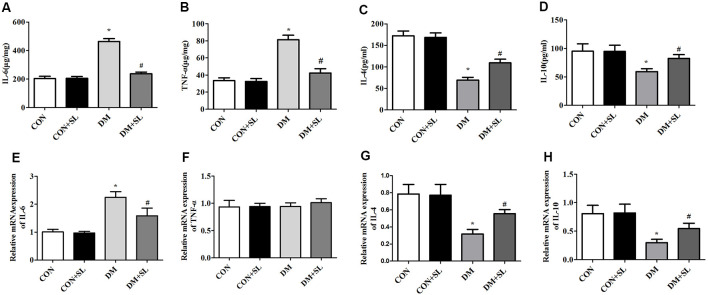
Effect of strawberry leaf extract on IL-6, TNF-α, IL-4, and IL-10 in the four groups. The data in each group are shown as the mean ± SD. **(A)** Expression of mIL-6. **(B)** Expression of mTNF-α. **(C)** Expression of mIL-4. **(D)** Expression of mIL-10. **(E)** Content of IL-6. **(F)** Content of TNF-α. **(G)** Content of IL-4. **(H)** Content of IL-10. *p < 0.05 vs. CON group; ^#^*p* < 0.01 vs. DM group, *n* = 5.

To assess the key roles of IL-6, TNF-α, IL-4, and IL-10 in mediating inflammation, ELISA was used to investigate the effect of SL on the secretion of these four cytokines on the hippocampus 4 weeks postadministration. Diabetes induced neuroinflammation in the hippocampus, with high levels of pro-inflammatory cytokines including IL-6 and TNF-α (*F* = 14.96, *p* < 0.05; *F* = 11.53, *p* < 0.05) and low levels of anti-inflammatory cytokines including IL-4 and IL-10 (*F* = 13.48, *p* < 0.05; *F* = 12.15, *p* < 0.05), and SL treatment decreased the levels of IL-6 and TNF-α (*F* = 11.35, *p* < 0.05; *F* = 14.82, *p* < 0.05; Figures 5A,B), and increased the levels of IL-4 and IL-10 in the hippocampus of the DM rats (*F* = 10.27, *p* < 0.05; *F* = 12.36, *p* < 0.05; Figures 5C,D). To elucidate the role of SL regulating these inflammatory cytokines in the hippocampus, we also detected mRNA levels of these four cytokines in the hippocampus with RT-PCR. The results showed that SL downregulated the mRNA levels of IL-6 in the rats with diabetes (*F* = 13.18, *p* < 0.05; Figure 5E) and up-regulated the mRNA levels of IL-4 and IL-10 (*F* = 9.98, *P* < 0.05, *F* = 10.75, *P* < 0.05; Figures 5G,H); however, there were no significant differences in the mRNA levels of TNF-α between the four groups (*F* = 1.32, *P* > 0.05; Figure 5F). Our results indicated that inflammatory factors make a great contribution to diabetic cognitive dysfunction.

### Strawberry Leaf Extract Mediated Cognitive Correlation Protein Expression

**Figure 6:**
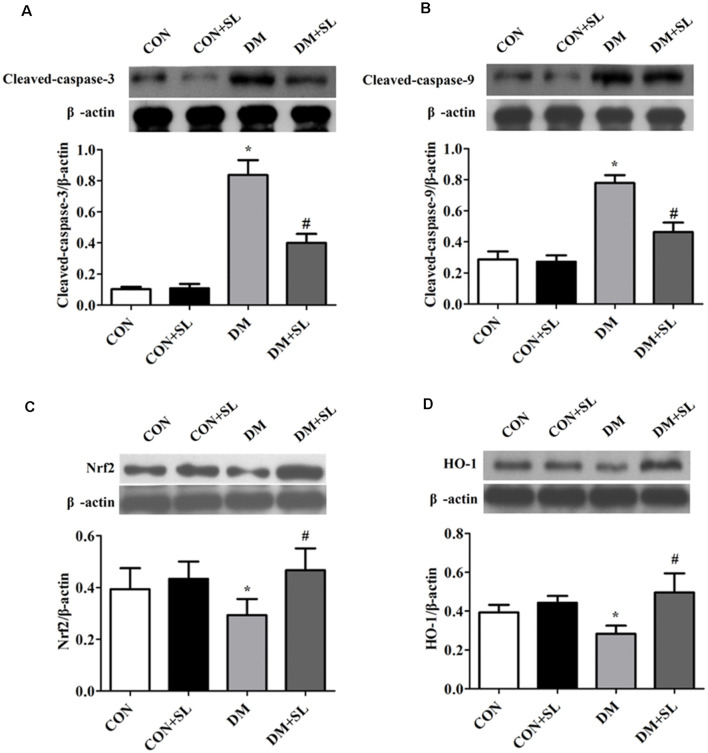
Effect of strawberry leaf extract on the expression of caspase-3, caspase-9, Nrf2, and HO-1. The protein of the five animals per group pulled and used in the electrophoresis, and then select a representative one. The results were the means of five tests. **(A–D)** Representative western blot images of caspase-3, caspase-9, Nrf2, and HO-1. The data in each group are shown as the mean ± SD. The values are the ratio of target proteins to β-actin. *p < 0.05 vs. CON group; ^#^*p* < 0.01 vs. DM group, *n* = 5.

In this experiment, we analyzed the expression of caspase-3, caspase-9, Nrf2, and HO-1 by Western blot. The results showed that hyperglycemia induced the upregulation of caspase-3 and caspase-9 (*F* = 23.95, *p* < 0.05; Figure 6A; *F* = 19.37, *p* < 0.05; Figure 6B), downregulation of Nrf2 and HO-1 (*F* = 21.58, *p* < 0.05; Figure 6C; *F* = 23.72, *p* < 0.05; Figure 6D). After 4 weeks of administration, SL reduced the expression of caspase-3 and caspase-9 (*F* = 19.15, *p* < 0.05; Figure 6A; *F* = 20.91, *p* < 0.05; Figure 6B), and increased the expression of Nrf2 and HO-1 in the DM rats (*F* = 17.39, *p* < 0.05; Figure 6C; *F* = 22.18, *p* < 0.05; Figure 6D). These results suggested that a high glucose environment in the rats may increase the expression of apoptosis-related proteins and impair the cognitive function of the rats. Apoptosis may be involved in diabetic cognitive dysfunction. Besides, we also observed changes in the proteins related to the Nrf2/HO-1 pathway, indicating that the Nrf2/OH-1 pathway is likely involved in diabetic cognitive dysfunction.

## Discussion

Diabetic cognitive dysfunction is a degenerative disease of the central nervous system. At present, no drug can effectively prevent the occurrence and development of diabetic cognitive dysfunction (Palta et al., 2019). The development of new drugs requires a large amount of economic investment and a long process. Therefore, clarifying the mechanisms of existing drugs and exploiting their precise and effective targets may be an effective approach for the treatment of diabetic cognitive dysfunction. Diabetes can raise blood levels and trigger oxidative stress and inflammation in diabetic rats (Nna et al., 2019). Our results indicate that strawberry leaf extract significantly alleviates cognitive and memory dysfunction in a diabetic animal model in SD rats.

The hippocampus is an important structure that is related to learning and memory in rats and humans, and the hippocampus plays an important integration role in learning and memory (Reaven et al., 1990). Therefore, the changes in the structure of the hippocampus caused by hyperglycemia may be the pathological basis of cognitive decline in diabetic rats (Bottino et al., 2002). In the present experiment, we found that through behavioral experiments that the diabetic rats showed impairments in learning and memory. The HE-stained hippocampal tissue sections showed that hippocampal neurons in the diabetic rats were damaged. Fortunately, after SL treatment, the blood sugar of the diabetic rats was significantly reduced, and the cognitive impairment and hippocampal neuronal damage were alleviated. Our results indicated that SL reduced the blood sugar of diabetic rats and improved their cognitive function.

SL reduced the cognitive decline in diabetic rats and may be a candidate for drugs to treat hypoglycemia and diabetic cognitive impairment. We tried to determine the underlying mechanisms of SL by examining some of the parameters associated with diabetes. Chronic hyperglycemia has been reported to induce an inflammatory response that produces inflammatory cytokines, such as TNF-α and IL-6 (Pickering et al., 2018; Mendoza-Núñez et al., 2019). These inflammatory cytokines are thought to contribute to diabetes-induced complications including cognitive impairment (Cunningham et al., 2009; Ceriello, 2003; Hall et al., 2013; El-Refaei et al., 2014). IL-4 is usually an anti-inflammatory cytokine secreted by activated Th2 cells, which can effectively prevent inflammatory cell infiltration and inflammatory cascade (Panayi and Corrigall, 2006). The main role of anti-inflammatory cytokines IL-10 is to inhibit the antigen presentation of monocyte macrophages and the release of inflammatory mediators, such as inhibiting the release of TNF-α and IL-6, and to maintain the balance of inflammatory cytokines in the body that is one of the important anti-inflammatory factors (Sabat et al., 2010). In this study, we found that chronic hyperglycemia could lead to a substantial secretion of TNF-α and IL-6 and little secretion of IL-4 and IL-10. After treatment with SL, this phenomenon was reversed. Also, the learning and memory impairment of the rats was significantly improved. Therefore, we hypothesized that SI could improve the cognitive function of diabetic rats by regulating the inflammatory response.

Moreover, ROS can directly damage islet β cells, promote β cell apoptosis, and indirectly inhibit β cell function by affecting the insulin signaling pathway (Kamata et al., 2005). Impaired beta cells, decreased insulin secretion levels, delayed secretion peaks, and increased blood glucose fluctuations make it difficult to control the rapid rise in postprandial blood glucose and cause more significant damage to cells (Tiedge et al., 1997). Clinical and basic studies have shown that oxidative stress is one of the important pathological mechanisms of diabetes (Das et al., 2019). Hyperglycemia is strongly associated with the development of cognitive impairment. Chronic hyperglycemia can lead to an excessive oxidative stress response, which is a key factor in dementia in diabetes (Tian et al., 2017). It has been reported that the overproduction of MDA and ROS and the reduction in SOD and CAT activities in different regions of the brain can lead to morphological abnormalities and memory loss (Qu et al., 2017). In our study, the data illustrated that strawberry leaf extract could inhibit excessive MDA production and enhance SOD and CAT activities. The results indicated that SL has a strong antioxidant effect, which can improve diabetes-associated cognitive dysfunction.

Apoptosis is induced by the activation of caspases. Caspases are effectors of apoptosis that can act with many substrates, leading to apoptosis and specific biochemical changes. Caspase-9 and caspase-3 become activated through a series of cascade reactions, eventually causing apoptosis (Hüttemann et al., 2011). In our study, we found that caspase-9 and caspase-3 were activated in diabetic rats; however, SL effectively inhibited their expression. Our findings showed that strawberry leaf extract could inhibit caspase cascade to reduce cognitive dysfunction in diabetic rats.

The relevant indicators of inflammation, oxidative stress, and apoptosis that we tested indicated that these processes may be involved in cognitive impairment in diabetes. Our next task was to identify the potential mechanisms. The Nrf2/HO-1 signaling pathway is the main regulator of the body’s antioxidative stress, protecting the body from the various pathological changes caused by oxidative stress. With the advancement of research, it has been found that this pathway has a wide range of functions in the human body, which are related to inflammation (Yu et al., 2014), aging (Prasad and Bondy, 2014), apoptosis (Bhakkiyalakshmi et al., 2014), nerve damage (Li et al., 2014), etc. When the Nrf2/HO-1 pathway is activated, it protects the body from the damage caused by inflammation, apoptosis, and oxidative stress. Our results show that Nrf2 and HO-1 were highly expressed in the diabetic rats after treatment with SL. Therefore, we assumed that the Nrf2/HO-1 pathway may be involved in diabetic cognitive dysfunction and that the activation of this pathway could effectively reduce the cognitive impairment caused by diabetes.

## Conclusion

SL can protect diabetic rats from cognitive dysfunction by activating the Nrf2/HO-1 signaling pathway and through anti-inflammatory, anti-apoptosis, inhibition of oxidative stress mechanisms. Elucidating the complex mechanism by which SL improves the conditions of diabetic rats provides a theoretical basis for the development of effective drugs that can improve the cognitive function associated with diabetes. Because the DACI rats in this study were in the stage of mild cognitive impairment, our findings may have guiding significance for the study of potential DACI therapies.

## Data Availability Statement

All datasets generated for this study are included in the article.

## Ethics Statement

The rat experiments were reviewed and approved by the Ethics Committee of the Second Affiliated Hospital of Soochow University.

## Author Contributions

YZ made substantial contributions to the conception and design. LZ drafted the manuscript. QM made contributions to the analysis and interpretation of data.

## Conflict of Interest

The authors declare that the research was conducted in the absence of any commercial or financial relationships that could be construed as a potential conflict of interest.
